# Elafin as a Predictive Biomarker of Acute Skin Graft-*Versus*-Host Disease After Haploidentical Stem Cell Transplantation Using Post-Transplant High-Dose Cyclophosphamide

**DOI:** 10.3389/fimmu.2021.516078

**Published:** 2021-02-19

**Authors:** Laura Solán, Diego Carbonell, Paula Muñiz, Nieves Dorado, Elena Landete, María Chicano-Lavilla, Javier Anguita, Jorge Gayoso, Mi Kwon, José Luis Díez-Martín, Carolina Martínez-Laperche, Ismael Buño

**Affiliations:** ^1^Department of Hematology, Gregorio Marañón General University Hospital, Madrid, Spain; ^2^Department of Translational Oncology, Gregorio Marañón Health Research Institute (IiSGM), Madrid, Spain; ^3^Department of Medicine, Complutense University of Madrid, Madrid, Spain; ^4^Genomics Unit, Gregorio Marañón Health Research Institute (IiSGM), Gregorio Marañón General University Hospital, Madrid, Spain; ^5^Department of Cell Biology, Complutense University of Madrid, Madrid, Spain

**Keywords:** haploidentical stem cell transplantation, skin graft *versus* host disease, prognostic biomarkers, elafin, high-dose cyclophosphamide

## Abstract

Haploidentical hematopoietic stem cell transplantation (haplo-HSCT) has shown favorable results in the treatment of hematological malignancies. Despite the use of post-transplant cyclophosphamide (PTCy), graft *versus* host disease (GVHD) remains as one of the main complications in this setting. Since the skin appears affected in up to 80% of cases of acute GVHD (aGVHD), its prognosis and diagnosis are essential for the correct management of these patients. Plasma concentration of elafin, an elastase inhibitor produced by keratinocytes, has been described elevated at the diagnosis of skin GVHD, correlated with the grade of GVHD, and associated with an increased risk of death. In this study we explored elafin plasma levels in the largest series reported of T cell–replete haplo-HSCT with PTCy. Plasma samples drawn from 87 patients at days +15 and +30 were analyzed (“discovery cohort”). Elafin levels at days +15 were no associated with chronic GVHD, non-relapse mortality, relapse, therapy-resistant GVHD, or overall survival. In our series, elafin levels at day +30 were not associated with post-transplant complications. On the other hand, elafin plasma levels at day +15 were higher in patients with severe skin aGVHD (21,313 *vs.*14,974 pg/ml; p = 0.01). Of note, patients with higher elafin plasma levels at day +15 presented a higher incidence of stage III-IV skin aGVHD (HR = 18.9; p < 0.001). These results were confirmed (HR = 20.6; p < 0.001) in an independent group of patients (n = 62), i.e. the “validation cohort.” These data suggest that measurement of elafin in patients undergoing haplo-HSCT with PTCy might be useful for an early identification of those patients who are at higher risk of suffering severe skin aGVHD and thus, improve their treatment and prognosis.

## Introduction

Haploidentical hematopoietic stem cell transplantation (haplo-HSCT) has shown favorable results in the treatment of hematological pathologies (1). Despite the use of post-transplant cyclophosphamide (PTCy), graft *versus* host disease (GVHD) remains as one of the main complications in this transplant setting (2, 3). The skin is involved in almost 80% cases of acute GVHD (aGVHD), and presentation can range from a limited maculopapular rash to wide skin involvement with ulcer formation (4). The diagnosis of skin aGVHD is based on clinical criteria and may be confirmed by skin histopathology, however, it has limitations in accurately differentiating between skin aGVHD and other causes of skin involvement such as viral rashes or pharmacological reactions (5). Thus, in the last few years, different biomarkers have been studied to enable the prognosis and diagnosis of aGVHD (6–9). One of the most widely studied biomarker associated with the diagnosis and prognosis of skin aGVHD is elafin (10).

Elafin, also known as peptidase inhibitor 3 or skin-derived antileukoprotease (SKALP), is an epithelial protein that is secreted by keratinocytes in response to IL-1 and TNFα. It is overexpressed in inflamed epidermis and absent in normal skin. Paczesny *et al*. analyzed plasma samples from 492 patients who received HSCT derived from bone marrow and described increased elafin plasma concentrations at the onset of skin aGVHD and its correlation with aGVHD severity. In the multivariate analysis, elafin levels also predicted non relapse mortality (NRM) and overall survival (OS) independently of the area of the skin rash (10). These results could not be confirmed by other study groups in which no relationship was found with elafin plasma levels and NRM or OS (8). Similarly, different groups have shown that tissue elafin is a useful immunohistochemical marker for the diagnosis and prognosis of skin GVHD (11, 12). Most of these studies were performed in HLA-identical or umbilical cord blood–based allogeneic HSCT. To our knowledge, only one study has been performed on patients receiving PTCy. Kanakry *et al*. (13) explored seven plasma-derived proteins including elafin in 58 HLA-haploidentical and 100 HLA-matched related or unrelated T cell–replete HSCT. Samples were collected 1, 2, 6, and 12 months after transplant. High elafin plasma levels were associated with the occurrence of NRM, but not with aGVHD development. In this context, our objective was to analyze plasma levels of elafin at days +15 and +30 after transplant and to correlate them with complications in a large cohort of patients who underwent unmanipulated haplo-HSCT with high-dose PTCy.

## Patients and Methods

### Patient Population

We retrospectively analyzed 110 consecutive patients who underwent haplo-HSCT between 2009 and 2016 at a single center (**Table 1**, “discovery cohort”). We excluded 23 cases, nine due to death before day +30 (secondary to progression or sepsis) and 14 due to lack of plasma samples. All 87 patients analyzed received PTCy 50 mg/kg/day (days +3, +4), mycophenolate mofetil, and cyclosporine as GVHD prophylaxis from day +5. Donor lymphocyte infusion (DLI) was performed in 10 patients who had mixed chimerism or minimal residual disease detected by molecular or immunophenotypic methods. Other three patients received CD34^+^ selected stem cell boosts.

**Table 1:** Clinical characteristics of patients and transplants included in the “discovery cohort” and in the “validation cohort.”

Characteristics	Discovery cohort n = 87	Validation cohort n = 62	*p value*
Recipient median age, years (range)	46 (16–66)	45 (20–69)	0.6
Recipient sex, Female/Male, n	25/62	25/37	0.1
Female donor/Male recipient, n (%)	28 (32)	18 (29)	0.6
Donor median age, years (range)	40 (14–68)	35 (14–65)	0.6
Primary malignancy, n (%)			0.2
Acute myeloid leukemia	28 (32)	21 (34)	
Hodgkin lymphoma	20 (23)	5 (8)	
Non-Hodgkin lymphoma	11 (13)	8 (13)	
Acute lymphoblastic leukemia	9 (10)	11 (17)	
Myelodysplastic syndrome	7 (8)	9 (14)	
Myelofibrosis	3 (3)	1 (2)	
Multiple myeloma	2 (2)	2 (3)	
Chronic lymphocytic leukemia	2 (2)	1 (2)	
Aplasia	1 (1)	3 (5)	
Others	4 (5)	1 (1)	
Disease risk index, n (%)			0.2
Very high + high	34 (39)	17 (27)	
Intermediate	50 (57)	37 (59)	
Low	2 (2)	5 (8)	
Not apply	1 (1)	3 (5)	
Pretransplant disease status, n (%)			0.02
Complete remission	46 (53)	38 (61)	
Partial remission	33 (38)	12 (19)	
Active disease	8 (10)	12 (19)	
Previous autologous transplant, n (%)	28 (32)	11 (17)	0.02
Previous allogeneic transplant, n (%)	10 (11)	6 (9)	0.7
Recipient/Donor CMV serostatus, n (%)			0.1
Matched	58 (67)	49 (79)	
Mismatched	26 (30)	13 (21)	
Missing	2 (2)	–	
Conditioning regimen intensity, n (%)			0.06
Myeloablative*	35 (40)	34 (55)	
Reduced intensity conditioning^	52 (60)	28 (45)	
Stem cell source, n (%)			0.01
Bone marrow	10 (12)	–	
Peripheral blood	77 (88)	62 (100)	
CD34^+^ cell dose infused, × 10^6^/kg, median (range)			0.01
Bone marrow	3.07 (1.07–4.73)	–	
Peripheral blood	5.34 (2.24–11.4)	6.8 (3.1–10.3)	

*Myeloablative conditioning regimen: Fludarabine 40 mg/m^2^ for 4 days and Busulfan 3.2 mg/kg for 3 or 4 days.

^Reduced intensity conditioning regimen: Fludarabine 30 mg/m^2^ for 4 days.

Cyclophosphamide 14.5 mg/kg on days −6 and −5 and Busulfan 3.2 mg/kg from day −3 for 1 or 2 days.

CMV, cytomegalovirus.

To confirm the results obtained from the analysis of the “discovery cohort,” elafin plasma levels on day +15 were measured in an independent ”validation cohort” with the same inclusion criteria. The “validation cohort” included 62 consecutive patients who underwent haplo-HSCT with PTCy between 2017 and 2019 and from which there was stored plasma sample available. GVHD prophylaxis was the same as that used in previous patients. Stem cell source was peripheral blood (PB) in all cases (**Table 1**, “validation cohort”).

### Definitions

NRM was defined as death not preceded by disease progression or relapse. Event-free survival (EFS) was defined as the time from transplantation to disease relapse or progression, re-transplantation due to graft failure, or death from any cause, whichever occurred first. OS was defined as the time from transplantation to death from any cause. aGVHD and chronic GVHD (cGVHD) were scored according to established criteria (14, 15). Steroid-resistant aGVHD was defined as progressive aGVHD after at least 3 days of methylprednisolone (2 mg/kg/day) or if unimproving grade III-IV aGVHD persisting after at least 7 days of initial treatment with methylprednisolone.

### Sample Collection and Processing

Samples from the 87 patients included in the “discovery cohort” were collected at days +15 and/or +30 after transplantation. All patients had at least one sample from one of the two timepoints. Elafin plasma levels were available on day +15 in 70 patients and on day +30 in 75 patients. Results for both plasma samples were available in 58 patients. Additionally, samples at day +15 were collected from 62 patients from the “validation cohort.” Plasma was obtained from blood samples by refrigerated (4°C) centrifugation at 2,000 rpm for 30 min in the 2–6 h following extraction. Samples were aliquoted without additives into cryovials and stored at −80°C. Elafin was detected using ELISA according to the manufacturer’s instructions (R&D Systems, USA). Samples (diluted 1/50) and standards were run in duplicate and absorbance was measured using the VICTOR2 D fluorometer™ (multilabel plate reader).

### Statistical Analysis

Numerical and categorical variables were expressed as median (range) and frequency (percentage), respectively. The Mann-Whitney test was used to compare differences between two independent variables. The determination of the best cut-off for elafin levels to stratify patients was derived from receiver operating characteristic (ROC) curves.

Competing risks were death, relapse, and DLI before +180 for aGVHD.

Univariate analyses were done using the log-rank test for EFS, and OS and Gray’s test for cumulative incidence. For the subanalysis carried out to study the relationship between elafin levels on day +30 and the appearance of aGVHD, those patients who had presented GVHD before day +30 were censored. OS and EFS were calculated using the Kaplan-Meier method. Statistical analyses were performed using SPSS v18 for Windows (SPSS Inc., Chicago, IL, USA). Cumulative incidence (CI) rates were calculated using the statistical package R ver. 3.3.2.

## Results

Baseline characteristics of 87 patients from the “discovery cohort” who underwent haplo-HSCT with PTCy are detailed in **Table 1**. The median follow-up period was 41 months (range, 15–109 months). Median age was 46 years (range, 16–66), and the most common stem cell source used was PB. CI of grade II-IV and grade III-IV aGVHD at 100 days was 51 and 14%, respectively. Likewise, the CI of stage II-IV and III-IV skin aGVHD at 100 days was 50 and 8%, respectively. Skin aGVHD has a median time to onset of 36 days (range 15–150 days). Twenty-four patients presented skin aGVHD before day 30. The CI of moderate-to-severe cGVHD at 2 years was 10%, and that of relapse and NRM at 2 years was 27 and 22%, respectively. Median time between haplo-HSCT and DLI was 253 days (range 55–645 days). Two-year OS and EFS were 62 and 50%, respectively.

No association was found between median elafin levels and clinical variables such as age, sex, stem cell source, donor sex, hematological malignancy, disease status at transplant, HSCT–associated comorbidity, previous transplant, conditioning regimen intensity, and number of infused CD34^+^ cells (data not shown).

We correlated median elafin levels at days +15 and +30 with post-transplant complications (**Table 2**). Median elafin levels on day +15 and on day +30 were 15,985 (range 843–22,119) and 15,375 (range 3,725–24,393), respectively. Elafin levels at day +30 were not associated with post-transplant complications. Moreover, we performed a subanalysis in which patients who presented aGVHD before day 30 were censored. Nevertheless, no correlation was either found with elafin levels at day +30 (data not shown).

**Table 2:** Association between elafin levels at day +15 and +30 and GVHD (acute and chronic), skin GVHD, NRM, relapse, and OS.

Whole cohort (n = 87)		Elafin +15 (pg/ml) Median (range)	*p value*	Elafin +30 (pg/ml) Median (range)	*p value*
**aGVHD II-IV**	YES NO	17,570 (4,888–22,120) n = 37 14,833 (843–21,449) n = 30	0.46	15,375 (5,254–24,393) n = 37 14,646 (3,543–22,449) N = 33	0.53
**aGVHD III-IV**	YES NO	19,324 (8,961–22,001) n = 12 14,794 (843–22,120) n = 55	0.1	9,721 (5,949–22,172) n = 10 15,039 (3,542–24,393) n = 60	0.22
**GI aGVHD** **II-IV**	YES NO	18,546 (9,920–21,336) n = 13 14,440 (843–22,120) n = 54	0.1	15,789 (6,922–21,586) n = 10 14,644 (3,541–24,393) n = 60	0.8
**Hepatic aGVHD II-IV**	YES NO	18,007 (8,960–20,382) n = 5 15,357 (843–22,119) n = 62	0.77	12,958 (7,893–17,866) n = 6 14,675 (3,541–24,393) n = 64	0.47
**Skin aGVHD II-IV**	YES NO	18,481 (4,888–22,120) n = 37 14,586 (843–21,449) n = 30	0.2	14,931 (5,254–24,393) n = 36 14,675 (3,542–22,449) n = 34	0.38
**Skin aGVHD III-IV**	YES NO	21,313 (14,014–22,001) n = 6 14,974 (843–22,120) n = 61	0.01*	15,442 (5,949–22,172) n = 7 14,646 (3,542–24,393) n = 63	0.9
**cGVHD** **(moderate and severe)**	YES NO	16,229 (843–20,837) n = 11 17,082 (4,888–22,120) n = 47	0.39	16,258 (7,893–21,811) n = 13 14,643 (3,542–24,393) n = 49	0.3
**Relapse**	YES NO	14,190 (7,515–21,449) n = 19 17,271 (843–22,120) n = 51	0.29	14,675 (3,542–22,449) n = 22 15,442 (3,725–24,393) n = 53	0.87
**Non-relapse mortality**	YES	19,082 (7,475–22,120)	0.19	14,675 (3,542–22,449)	0.16
		n = 15		n = 16	
	NO	16,229 (843–22,001)	0.19	14,278 (4,596–24,393)	0.16
		n = 41		n = 41	
**Status at last follow-up**	Dead Alive	14,400 (7,475–22,120) n = 29 16,229 (843–22,001) n = 41	0.64	16,393 (3,542–21,949) n= 34 14,278 (4,596–24,393) n = 41	0.2
		

aGVHD, acute graft vs host disease; cGVHD, chronic graft vs host disease; GI, gastrointestinal; *Statistically significant.

We did not find a relationship between elafin levels at days +15 and +30 and the development of therapy-resistant GVHD.

**Figure 1:**
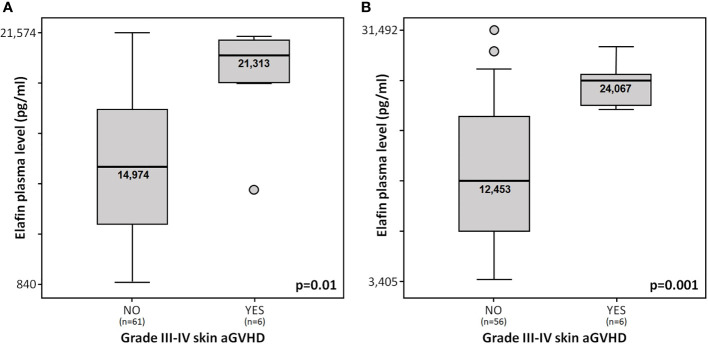
**(A)** Elafin plasma levels at day +15 in patients from “discovery cohort” with and without stage III-IV skin aGVHD. **(B)** Elafin plasma levels at day +15 in patients from the “validation cohort” with and without stage III-IV skin aGVHD.

Elafin levels at day +15 were not associated with cGVHD, NRM, relapse or OS. Instead, at day +15, median elafin levels seemed higher in patients with grade II-IV and III-IV aGVHD compared with those without GVHD (17,570 *vs.* 14,833 pg/ml; p = 0.46 and 19,324 *vs.* 14,794; p = 0.1, respectively), with statistically significant differences in those with severe skin involvement (21,313 *vs.*14,974 pg/ml; p = 0.01; **Figure 1A**).

**Figure 2:**
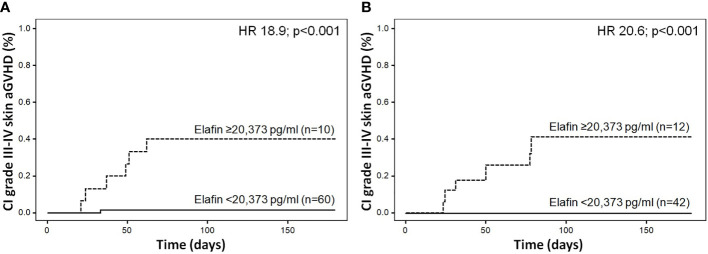
**(A)** Cumulative incidence of stage III-IV skin aGVHD according to elafin plasma levels at day +15 in the “discovery cohort”. **(B)** Cumulative incidence of stage III-IV skin aGVHD according to elafin plasma levels at day +15 in the “validation cohort.”

ROC curve analysis revealed that the best cut-off value for elafin levels at day +15 for stage III-IV skin aGVHD was 20,373 pg/ml. Patients with elafin levels higher than 20,373 pg/ml at day +15 presented a significantly higher incidence of stage III-IV skin aGVHD (HR = 18.9; p < 0.001; **Figure 2A**). We were unable to find an optimal cut-off elafin level to stratify patients correctly regarding development of grade II-IV and III-IV aGVHD (data not shown).

Elafin levels at day +15 were significantly associated with greater CI of stage III-IV skin aGVHD (subdistribution hazard ratio, SHR = 26.5; p = 0.003). No association was found for the other variables analyzed (**Table 3**).

**Table 3:** Univariable associations between elafin levels at day +15 and clinical variables with stage III-IV skin acute GVHD using the Fine-Gray model.

Variables	Skin aGVHD III-IV	
	SHR (95% CI)	*p-value*
Age >50 years	0.62 (0.12–3.23)	0.5
Female sex	1.03 (0.19–5.43)	0.96
Sorror >3	0.51 (0.09–2.67)	0.4
DRI Very high + high Previous HSCT	1.14 (0.25–5.08) 1.35 (0.15–11.98)	0.81 0.78
Underlying disease other than AML	7.86 (0.91–67.39)	0.06
Infused TNC >6 × 10^8^/kg	0.98 (0.19–5.06)	0.98
RIC conditioning regimen	1.67 (0.32–8.66)	0.5
Elafin +15 ≥20,373 pg/ml	26.5 (3.14–223.3)	0.003*

SHR, subdistribution hazard ratio; HSCT, hematopoietic stem cell transplantation; AML, acute myeloid leukemia; TNC, total nucleated cells; DRI, disease risk index; *Statistically significant.

Elafin levels were also analyzed on day +15 in an independent “validation cohort” (n = 62). Both cohorts of patients were homogeneous except for the pretransplant disease status, the proportion of patients who had previously undergone autologous transplantation, the stem cell source, and the amount of PB CD34^+^ cells infused (**Table 1**). Six patients presented stage III-IV skin aGVHD. Skin aGVHD showed a median time to onset of 39 days (range 22–78 days). Median elafin levels were significantly higher in patients with stage III-IV skin aGVHD compared with those without GVHD (24,067 *vs.* 12,453 pg/ml; p = 0.001; **Figure 1B**).

Patients with elafin levels higher than 20,373 pg/ml at day +15 presented a significantly higher incidence of stage III-IV skin aGVHD (HR = 20.6; p < 0.001). In addition, all patients with stage III-IV skin aGVHD had elafin levels on day +15 greater than 20,373 pg/ml (**Figure 2B**).

## Discussion

Despite the proven efficacy of PTCy as GVHD prophylaxis in haplo-HSCT, GVHD remains as one of the main causes of NRM and may have a significant negative impact on the patient’s quality of life (1). Many different organs can be involved, which leads to a wide range of clinical manifestations. Skin, gut, and liver are the major target organs in aGVHD, and therefore the classic symptoms of rash, diarrhea, and elevated bilirubin levels strongly suggest the diagnosis. In this context, the skin is involved in almost 80% cases of aGVHD (12). Cutaneous manifestations are described as erythematous maculopapular morbilliform eruptions starting on the face, ears, palms, and soles. Follicular erythema is a frequent aGVHD early manifestation, and both erythematous macular and papular rashes can occur (16). In spite of such signs, GVHD diagnosis can be confusing since other etiologies such as the drug hypersensitivity reaction or viral exanthems, can appear with the same symptomatology. The diagnosis of skin aGVHD is presently based on clinical criteria and is supported by histopathology. There is growing evidence about limitations of skin histopathology for definitive GVHD diagnosis (5, 17). In this context, in the last decade several biomarkers have been described in order to improve clinical and histopathological diagnosis, prediction of disease occurrence, and response to therapy (6–13). Despite their proven usefulness, they are not yet part of the routine clinical practice.

Most studies in this regard have been performed on HLA-identical or umbilical cord blood allo-HSCT. Only one study analyzed haplo-HSCT with PTCy (13). In the present study, we explored plasma levels of elafin in the largest single-center cohort of haplo-HSCT with PTCy investigated to date.

The median levels of elafin on day +15 were, in general, higher than those on day +30 in patients who presented GVHD. Similar results were obtained with elafin values in other studies (8, 18). The median elafin levels obtained in our study were similar to those obtained in the haplo-HSCT cohort of the Baltimore group (13). Conversely, the median values of elafin found by other groups (8, 10, 18) were lower. This could be explained by the use of different donor types, conditioning regimen intensity or GVHD prophylaxis. Moreover, laboratory testing details, such as the dilution of the sample or the ELISA technique for the detection of elafin, were not homogeneous throughout the different reports. In this regard, in future studies it would be important to consider unifying or centralizing the commercial ELISA kits used, the sample (plasma or serum), and the processing of the sample (fresh or frozen). Also, our results show that elafin levels at day +15 were higher in patients who presented with grade II-IV and III-IV aGVHD compared with those without GVHD, reaching statistical significance in those who presented stage III-IV skin aGHVD (p = 0.01). In order to validate our results, we have included the Fine-Gray model to directly estimate the effect of elafin on the cumulative incidence function of the outcome (in the presence of competing risks). We confirmed that elafin levels at day +15 were significantly associated with greater CI of stage III-IV skin aGVHD (SHR = 26.5; p = 0.003). We also analyzed elafin plasma levels on day +15 in an independent “validation cohort.” Once again, elafin levels were significantly higher in patients with severe skin aGVHD compared with patients without skin aGVHD (p = 0.001). These results would confirm the predictive role of elafin levels on day +15 for severe skin aGVHD in haplo-HSCT.

Consistent with our study, Paczesny *et al*. (10) analyzed a total of 492 patients who received a HSCT and described higher elafin plasma levels at the time of diagnosis of skin GVHD which correlated with greater stages of skin GVHD. However, this study was not fully comparable with ours, since in their cohort all patients received bone marrow as stem cell source and elafin levels were measured at the time of diagnosis and not in advance.

Results of the only study performed on haplo-HSCT (13) differed from those of the present study. The authors did not find any relationship between elafin levels and the appearance of GVHD. Unlike ours, a high proportion of patients with bone marrow as stem cell source were included, therefore, the number of patients presenting grade II-IV aGVHD in their cohort was lower (n = 10). Furthermore, the first post-transplant plasma sample were drawn at day +30, and patients with the GVHD onset prior to that timepoint were excluded from the analysis. Therefore, algorithms to assign specific timepoints for intervention will need to be established, ideally in prospective multicenter trials.

In our study, elafin plasma levels were not associated with NRM or OS. Some studies did not find either a statistically significant association between these entities (8), whereas others observed a correlation between elafin plasma levels and NRM (10, 13). In our cohort, GVHD itself justifies half of the transplantation related deaths of which stage III-IV skin aGVHD is only present in two patients. This fact and the relatively small simple size and that in our cohort, might not yield sufficient statistical power to detect the prognostic value for mortality of this biomarker.

Our analysis is subject to a number of limitations. We collected plasma samples on days +15 and +30 after transplant. Most probably, the most appropriate samples for the clinical outcomes assessed should be collected earlier and more frequently after transplant, besides at the onset of GVHD and in the following months. Such an approach could prove crucial for future proteomic biomarker studies.

Despite the low number of patients included in the present study, which could be seen as a limitation, the apparent strength of the association between high levels of elafin on day +15 and the consequent onset of stage III-IV skin aGVHD made it able to be uncovered. In order to confirm our results, we have included the Fine-Gray model to directly estimate the effect elafin on the cumulative incidence function of the outcome (in the presence of competing risks) and we have performed the measurement of elafin levels on day +15 in an independent “validation cohort.” Although the latter confirmed our results, the use of biomarkers in routine clinical practice should be validated in a larger cohort in a prospective multicenter study.

Even with the need for further research, our work supports the fact that elafin is a valid plasma biomarker in the setting of haplo-HSCT with PTCy. Elafin could have a predictive role for the development of severe skin aGVHD. The results reported here could provide the basis for future clinical trials to analyze, in a controlled way, the optimal modulation of the immunosuppressive treatment in this particular transplant setting.

## Data Availability Statement

All datasets generated for this study are included in the article/supplementary material.

## Ethics Statement

The studies involving human participants were reviewed and approved by Comité de Ética de la Investigación con Medicamentos (CEIm) del Hosp. G.U. Gregorio Marañón. The patients/participants provided their written informed consent to participate in this study.

## Author Contributions

Conception and design: LS, CM-L, IB. Data collection: PM, ND, EL, JA. Data analysis: LS, MK, DC, MC-L, JG, JD-M, CM-L, IB. Manuscript drafting: LS, CM-L, IB. Critical manuscript revision: all authors. All authors contributed to the article and approved the submitted version.

## Funding

The present study was partially supported by the Ministry of Economy and Competitiveness ISCIII-FIS (grants PI17/01880 and PI20/00521), co-financed by ERDF (FEDER) funds from the European Commission, “A way of making Europe,” as well as grants from the Asociación Madrileña de Hematología y Hemoterapia (AMHH).

## Conflict of Interest

The authors declare that the research was conducted in the absence of any commercial or financial relationships that could be construed as a potential conflict of interest.
